# Efficacy of cell-free DNA as a diagnostic biomarker in breast cancer patients

**DOI:** 10.1038/s41598-023-42726-6

**Published:** 2023-09-15

**Authors:** Iqra Khurram, Muhammad Umer Khan, Saooda Ibrahim, Ayman Saleem, Zaman Khan, Muhammad Mubeen, Arooj Khawar, Qurban Ali

**Affiliations:** 1 Faculty of Allied Health Sciences, University Institute of Medical Lab Technology, The University of Lahore, Lahore, Pakistan; 2 Institute of Molecular Biology and Biotechnology, The University of Lahore, Lahore, Pakistan; 3 Department of Plant Breeding and Genetics, Faculty of Agricultural Sciences, University of the Punjab, Lahore, Pakistan

**Keywords:** Biotechnology, Cancer

## Abstract

Breast cancer is the most prevalent and leading cause of mortality worldwide among women. Cell-free DNA (cfDNA) analysis is an alternative quantitative approach to conventional methods for cancer diagnosis. The current research project aimed to determine the efficacy of cfDNA as a diagnostic biomarker in breast cancer patients in Pakistan. Eighty-four female breast cancer patients were selected as cases, and 152 healthy females as controls. Immunohistochemistry was performed to identify tumor biomarkers along with clinical profiling. cfDNA was extracted from serum using the phenol–chloroform method. The cfDNA level in the serum was estimated using Agarose Gel Electrophoresis and Nanodrop. SPPS version 25.0 was used to perform statistical analyses. The results showed that the cancer biomarkers were significantly associated with breast cancer. The changes in hematological parameters were insignificant, whereas the biochemical parameter variations between the cases and controls were statistically significant. A significant association of cfDNA level with breast cancer was observed. Further cfDNA levels and cancer biomarkers were not statistically significant. A significant correlation was observed between cfDNA and biochemical parameters, except for creatinine, whereas hematological parameters showed no significant correlation.ROC analysis declared cfDNA as an authentic diagnostic marker for breast cancer. It was concluded that the level of cfDNA is significantly increased in breast cancer patients and can be utilized as a diagnostic biomarker.

## Introduction

Breast cancer is a complex and diverse disease affecting millions of women around the globe. It is the second leading cause of cancer-related deaths in women, mainly due to metastasis to organs such as the lungs, liver, bones, and brain^1^. The disease is stratified into molecular subtypes based on the presence of receptors which include: estrogen receptor (ER), progesterone receptor (PR), and human epidermal growth factor receptor 2 (HER2)^2^. Breast tumors with detectable ER, PR, or both, with or without HER2 amplification, are classified as luminal-like tumors^3^. Tumors that show HER2 overexpression but lack ER and PR expression are HER2 + breast cancers^4^. Triple-negative breast cancer (TNBC) lacks expression of all three receptors^5^. The role of the androgen receptor as a therapeutic target remains debated, although it is expressed in 60–90% of breast cancers^6^.

Breast cancer is a major cause of cancer-related deaths, with around 2.3 million new cases and approximately 650,000 deaths annually^7^. It is considered the most common cancer and the leading cause of female cancer death, particularly the hormone receptor-positive and HER2-negative subtypes^7^. In 2020, breast cancer accounted for approximately 11.7% of cancer cases and nearly 685,000 deaths worldwide^7^. According to the American Cancer Society, the US will have approximately 287,850 new breast cancer cases and 43,250 deaths by 2022^8^. Breast cancer diagnosis involves physical examination, imaging (e.g., mammography), tissue biopsy, and blood tests for specific antigens/proteins^9,10^. Early detection is critical for improving survival rates, highlighting the need for more sensitive and breast cancer-specific biomarkers to detect aggressive forms of the disease^11^.

Despite advances in breast cancer diagnosis and treatment, standard methods have limitations such as invasiveness, high cost, and low sensitivity and specificity, making them inappropriate for all patients^12^. Classical diagnostic techniques, including imaging and tissue biopsy, have drawbacks such as false-positive and false-negative results, radiation exposure, and inadequate representation of the tumor genomic landscape^13–15^. Although invasive tissue biopsy may not fully cover the molecular landscape of breast tumors and is insufficient for monitoring treatment responses^12^. Therefore, a better noninvasive technique for early detection, survival prediction, and treatment monitoring is urgently needed.

Circulating biomarkers, such as cell-free DNA (cfDNA), are gaining popularity as non-invasive options for disease monitoring^16,17^. cfDNA has emerged as a potential biomarker for various cancers (lung, breast, liver, etc.) owing to increased cellular processes such as apoptosis, necrosis, and autophagy in cancer patients^18^. Detecting changes in cfDNA levels in serum or plasma and identifying genetic abnormalities released by tumors holds promise for cancer diagnosis using liquid biopsy^18,19^. Tumor cells release cfDNA, known as circulating tumor DNA (ctDNA), and its elevated levels distinguish cancer patients from healthy individuals^19,20^. Recently, cfDNA was detected in serum, plasma, urine, and other fluids, offering insights into specific diseases^21^. As cfDNA is released from tumor sites, it is a surrogate marker for tissue biopsies, enabling rapid, noninvasive, and sensitive cancer diagnosis, prognosis, and therapy monitoring for different cancer types^22,23^. Moreover, cfDNA integrity (cfDI), which measures cfDNA fragmentation, holds promise as a diagnostic and prognostic biomarker^24^.

Elevated levels of cfDNA in cancer cells are attributed to reduced DNase activity. This increase in plasma cfDNA levels has been suggested for breast cancer diagnosis, with qualitative tests showing increased cfDNA integrity/size. However, elevated cfDNA levels can also be observed in benign breast diseases, reducing its specificity for cancer detection. Specific markers such as mutations, heterozygosity (LOH) loss, and hypermethylation in cfDNA patterns have been explored^25^. Various detection strategies for breast cancer using cfDNA include measuring cfDNA concentration, integrity, microsatellite alterations, gene mutations, and DNA methylation. The concentration of cfDNA is a quantitative approach and an initial detection strategy for breast cancer^26^.

Cheng et al. explored cfDNA variables as prognostic markers for metastatic breast cancer (MBC). The results showed increased cfDNA integrity (cfDI) and decreased cfDNA concentration after one therapy cycle with repetitive DNA elements. Both cfDI and cfDNA concentrations independently served as prognostic markers at the baseline and after therapy. Combining cfDNA variables with other markers enhanced the prognostic power in MBC patients, indicating their potential as promising prognostic markers during systematic therapy^27^.

This study compared cfDNA and CTCs with conventional breast cancer blood biomarkers in 194 patients (MBC). Total cfDNA levels were associated with progression-free survival (PFS), overall survival (OS), and therapy response, suggesting their potential as cost-effective blood-based tests for monitoring MBC patients. Combining CTCs and cfDNA enhances OS's predictive value compared to traditional biomarkers^28^.

Another study used cfDNA as a biomarker for the diagnosis of breast cancer. Quantitative PCR in 110 breast cancer patients, 95 benign tumor patients, and 90 healthy volunteers revealed higher cfDNA levels in patients with breast cancer. After chemotherapy, the cfDNA concentration and integrity decreased significantly. Correlation analysis linked cfDNA concentration to CEA and CA15-3, and cfDNA integrity to CA125. cfDNA showed higher sensitivity and specificity than traditional biomarkers, indicating its potential for distinguishing breast cancer from benign tumors and healthy individuals^29^.

The current investigation aims to explore the efficacy of cfDNA as a diagnostic biomarker for patients with breast cancer in Pakistan by assessing the association between cfDNA levels and breast cancer and examining the correlation between cfDNA levels and cancer biomarkers, hematological, and biochemical parameters.

## Material and methods

### Study population

A study conducted on 150 female patients with breast cancer revealed that 84 were diagnosed with cancer through a strut biopsy. Participants aged 18–40 years with primary breast cancer and no distant metastases were included, with written informed consent. The control group comprised 152 healthy women aged 18–40. The control group included healthy women examined alongside the experimental group without any lumps, trauma, inflammation, or cancer history. They should have comparable ages and baseline clinical data to those of patients with breast cancer and must provide voluntary consent. The exclusion criteria for the case and control groups were age < 18 or > 40 years, pregnancy, autoimmune diseases, organ transplantation, and ischemic stroke or brain hemorrhage. Six milliliters of blood were collected per participant, with 3 mL in an EDTA vial for whole blood and 3 mL in a Silicon gel vial for serum. Serum samples were stored at room temperature and transferred to the research laboratory within 3–4 h. After centrifuging the blood at 3000 RPM for 10 min, the serum was retrieved from the upper portion of the supernatant and stored in aliquots at -80℃. The Sysmex XP-300 automated analyzer was used for Complete blood count (CBC) and Roche Cobas c-311 for liver and renal function tests. This study was approved by the Ethical Review Board (ERB), reference number:305/18/08/2022/S1 ERB, The Jinnah Hospital Lahore, and was conducted by the international guidelines of the Declaration of Helsinki.

### Immunohistochemical analysis

A trucut biopsy extracted breast cancer tissue, then fixed in 10% neutral-buffered formalin. Dehydration and paraffin embedding were followed by heat-induced epitope retrieval for antigen unmasking on 4–5 m thick tissue sections. Next, endogenous peroxidase activity was inhibited, and non-specific binding was stopped using hydrogen peroxide as a blocking agent. After incubating the tissue sections with primary antibodies directed against the desired proteins, these were treated with the corresponding secondary antibody. A chromogenic substrate, 3,3'-diaminobenzidine (DAB), was utilized to observe the antibody-antigen complex, while counterstaining was performed to enhance tissue morphology. Both positive and negative controls were used for validation. Digital images of stained tissue slices were taken and examined to determine the staining intensity and distribution. Observers conducted data interpretation in a blinded manner, and statistical analysis was performed when applicable. The Allred scoring system assessed the expression of the HER2, ER/PR protein in breast cancer tissue samples.

### cfDNA extraction and estimation

The in-house phenol–chloroform method was used for cfDNA extraction from 500 µL serum samples. For digestion of the remnants, proteinase K, TNE buffer, and SDS were added and incubated at 37 °C, followed by precipitation with NaCl. After centrifugation, PCI was added to separate the upper aqueous layer from the organic layer that contained impurities. The cfDNA supernatant was then transferred to another Eppendorf tube and precipitated using chilled isopropanol and the resulting pellets were saved after centrifugation. After washing with 70% ethanol, cfDNA was dissolved in low TE buffer, placed in a 70 °C water bath for 1 h to deactivate enzymes, and stored at − 40 °C for preservation. Qualitative analysis of cfDNA was performed by agarose gel electrophoresis to determine its size. The Invitrogen DNA ladder was used as a reference. The extracted cfDNA was quantified using a Thermo Scientific Nanodrop One C based on spectrophotometric measurements at 260 nm.

### Statistical analysis

SPSS (version 25.0) was used for statistical calculations. The mean and standard deviation were used to quantify the cfDNA data, and the Student’s *t* test was used to compare continuous variables between breast cancer patients and healthy controls. Chi-square and Univariate logistic regression analyses were done to determine the association between cfDNA levels and breast cancer biomarkers. Spearman’s and Pearson's correlations were used to analyze the relationship between quantitative cfDNA levels and clinical markers. Receiver Operating Characteristic (ROC) analysis determines AUC, optimal cut-off value, and sensitivity calculated by Youden index. Statistically significant *P* values were < 0.05.

### Ethical approval

It has been confirmed that the experimental data collection complied with relevant institutional, national, and international guidelines and legislation with appropriate permissions from the Ethical Review Committee, University Institute of Medical Lab Technology, Faculty of Allied Health Sciences, The University of Lahore, Lahore, Pakistan.

## Results

**Figure 1:**
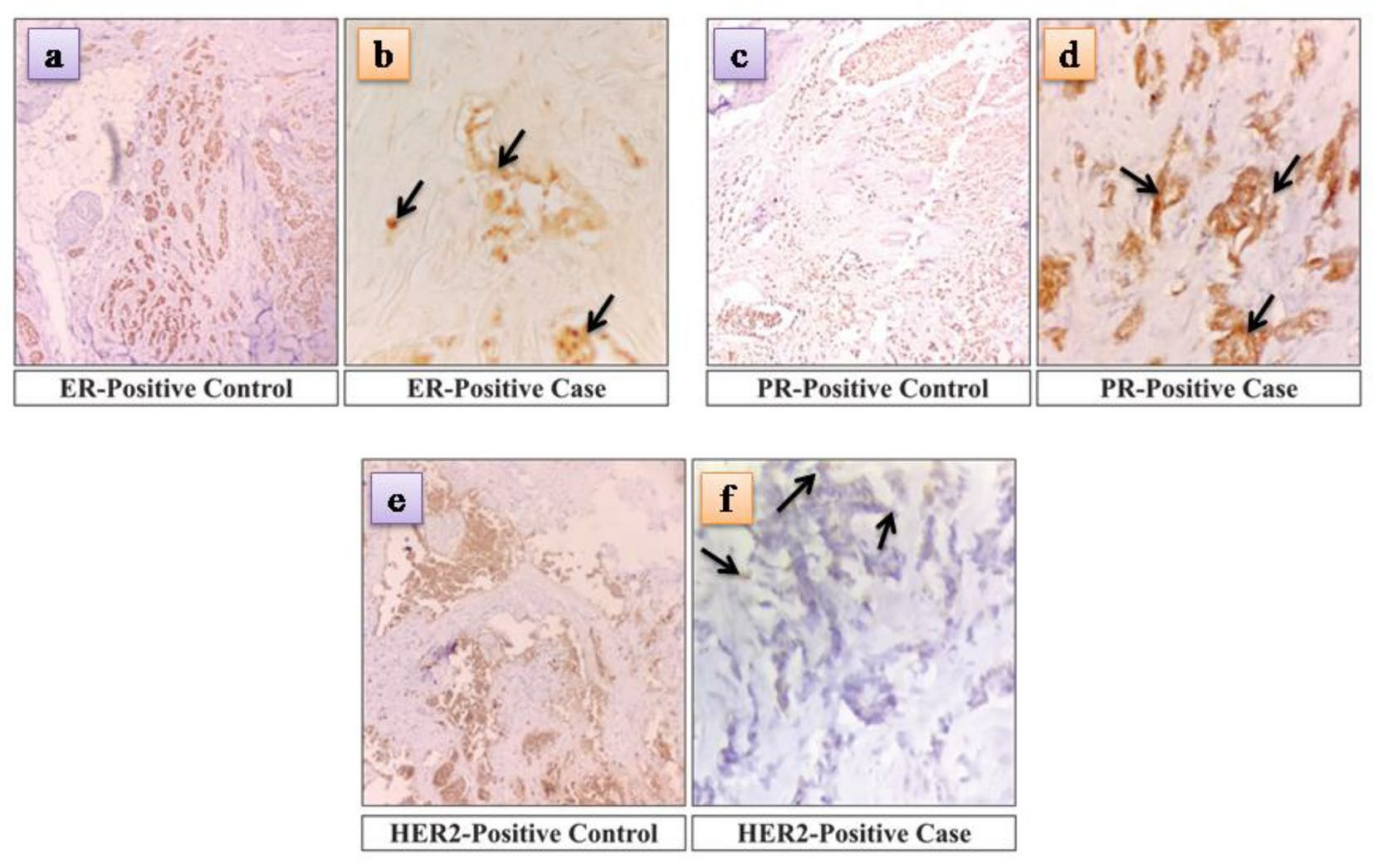
Microscopic examination of ER, PR, HER2 status in breast tissue core biopsy.

In this study, the criteria for diagnosing breast cancer as cases were Trucut biopsy and immunohistochemistry markers (IHC) specifically used for diagnosing breast cancer. A trucut biopsy of a patient with breast cancer revealed strong nuclear staining in more than 80% of tumor cells, an intensity score of 3, and a proportion score of 5, for a total Allred score of 8/8, indicating an ER-positive case (Fig. 1a,b). Similarly, strong nuclear staining was observed in 80% of the tumor cells, with an intensity score of 3 and a proportion score of 5, resulting in an Allred score of 8/8, indicating a PR-positive case (Fig. 1c,d). to 3–5% of the cells showed intense membrane staining, indicating a HER2-positive case (Fig. 1e,f).

### Baseline demographic characteristics of the subject

The current study included 84 patients with primary breast cancer selected as cases, and 152 healthy individuals taken as controls (shown in Table 1).

**Table 1:** Baseline characteristics of the sample population.

Characteristics	BC cases n = 84	Healthy controls n = 150
Age	18–40 years	18–40 years
ER		
Positive	51	–
Negative	33	
PR		
Positive	45	–
Negative	39	
HER2		
Positive	39	–
Negative	45	
TNBC		
Positive	13	–
Negative	71	
TPBC		
Positive	13	–
Negative	71	

### Baseline clinicopathological characteristics of subjects

Student *t* tests confirmed that the serum cfDNA levels in breast cancer patients were significantly greater (*p* ≤ 0.001) than in healthy controls (437.44.1). The hematological parameters did not show statistically significant differences between cases and controls, whereas biochemical parameters showed significant results. The cancer biomarkers HER2, ER, PR, TNBC, and TPBC were significantly associated with breast cancer patients. The clinicopathological characteristics of the study population are shown in Table 2.

**Table 2:** Clinicopathological characteristics of the subject.

Variables	BC patients (Mean ± SD)	Healthy control (Mean ± SD)	*P* value
Number	84	152	
Serum DNA yield (ng/µl)	1482 ± 1.7	437.4 ± 4.1	< 0.001
Hb	11.4 ± 1.4	21.2 ± 1.0	0.25
WBCs	7.2 ± 2.6	7.4 ± 0.8	0.41
PLTs	302.7 ± 1.3	295.3 ± 7.6	0.59
Urea	26.3 ± 7.5	16.2 ± 4.4	< 0.001
Creatinine	0.76 ± 0.2	0.69 ± 0.1	< 0.001
T. Bilirubin	52 ± 0.2	0.75 ± 0.2	< 0.001
ALT	41.5 ± 4.1	24.2 ± 5.9	< 0.001
AST	40.6 ± 3.4	23.4 ± 0.8	< 0.001
ALP	142.1 ± 0.8	111.6 ± 0.2	< 0.001
HER2 n(%)			
Positive	39 (46.4)	0 (0)	< 0.001
Negative	45 (53.6)	152 (100)	
ER n(%)			
Positive	51 (60.7)	0 (0)	< 0.001
Negative	33 (39.3	152 (100)	
PR n(%)			
Positive	45 (53.6)	0 (0)	< 0.001
Negative	39 (46.4)	152 (100)	
TNBC n(%)			
Positive	13 (15.5)	0 (0)	< 0.001
Negative	71 (31.8)	152 (100)	
TPBC n(%)			
Positive	13 (15.5)	0 (0)	< 0.001
Negative	71 (84.5)	152 (100)	

### Association of cfDNA with breast cancer

A chi-square test was applied to determine the utility of cfDNA as a valuable biomarker for breast cancer. These results indicate that cfDNA levels are statistically significant with BC, suggesting that cfDNA can be potentially utilized as a diagnostic and prognostic biomarker in breast cancer. Table 3 indicates the association between cfDNA levels and breast cancer.

**Table 3:** Association between cfDNA levels and breast cancer.

	Patients n(%)	Healthy control n(%)	AOR (C.I.)	*P* value
cfDNA				
< 1000 ng/µl	26 (31)	137 (90.1)	Reference	< 0.001
> 1000 ng/µl	58 (69.0)	15 (9.9)	1.003 (1.003–1.004)	

*C.I.* confidence interval.

*Correlation is significant at the 0.01 level (2-tailed).

### Association of cfDNA with cancer biomarkers

Univariate logistic regression analysis revealed that the levels of cfDNA quantified using Nanodrop analysis showed no significant association between cfDNA levels and cancer biomarkers. Table 4 briefly indicates the association of cfDNA with cancer biomarkers.

**Table 4:** Association of cfDNA with cancer biomarkers.

Cancer markers	cfDNA		*P* value
HER2 status	< 1000 ng/µl	> 1000 ng/µl	
Positive	13 (33.3)	26 (66.7)	0.66
Negative	13 (28.8)	32 (71.2)	
ER status			
Positive	14 (27.5)	37 (72.5)	0.38
Negative	12 (36.4)	21 (63.6)	
PR status			
Positive	10 (22.2)	35 (77.8)	0.06
Negative	16 (41)	23 (59)	
TNBC status			
Positive	5 (38.5)	8 (61.5)	0.52
Negative	21 (29.6)	50 (70.4)	
TPBC status			
Positive	3 (23.1)	10 (76.9)	0.5
Negative	23 (32.4)	48 (67.6)	

### Correlation of quantitative levels of cfDNA with clinical parameters

Pearson’s correlation between quantitative levels of cfDNA and clinical parameters indicated that hematological parameters showed a negative correlation with cfDNA. Further results indicate that the RFT and LFT parameters showed a positive correlation, except for bilirubin, with a significance value < 0.001, as illustrated in Table 5 (supplementary Figure 1).

**Table 5:** Pearson's correlation of clinical parameters with cfDNA in BC patients.

Variables	Pearson’s correlation coefficient (r)	*P* value
Hb	− 0.072	0.26
WBCs	− 0.029	0.64
PLTs	− 0.028	0.66
Urea	0.445	> 0.001
Creatinine	0.11	0.07
T. Bilirubin	− 0.217	0.001
ALT	0.282	< 0.001
AST	0.301	< 0.001
ALP	0.297	< 0.001

### Correlation of quantitative levels of cfDNA with cancer biomarkers

Spearman’s correlation between quantitative levels of cfDNA with cancer biomarkers indicated that cfDNA was significantly correlated with ER, PR, TNBC, and TPBC, except HER2, suggesting that cfDNA could be used as a diagnostic and prognostic biomarker for BC. Table 6 indicates Spearman’s correlation between cfDNA and cancer biomarkers in patients with BC.

**Table 6:** Spearman’s correlation of cfDNA with the cancer biomarkers in breast cancer patients.

Variables	Spearman’s coefficient (ρ)	*P* value
HER2	0.371	4.1
ER	0.518	< 0.001
PR	0.481	< 0.001
TNBC	0.227	< 0001
TPBC	0.21	0.001

### Receiver operative curve (ROC) analysis

**Figure 2:**
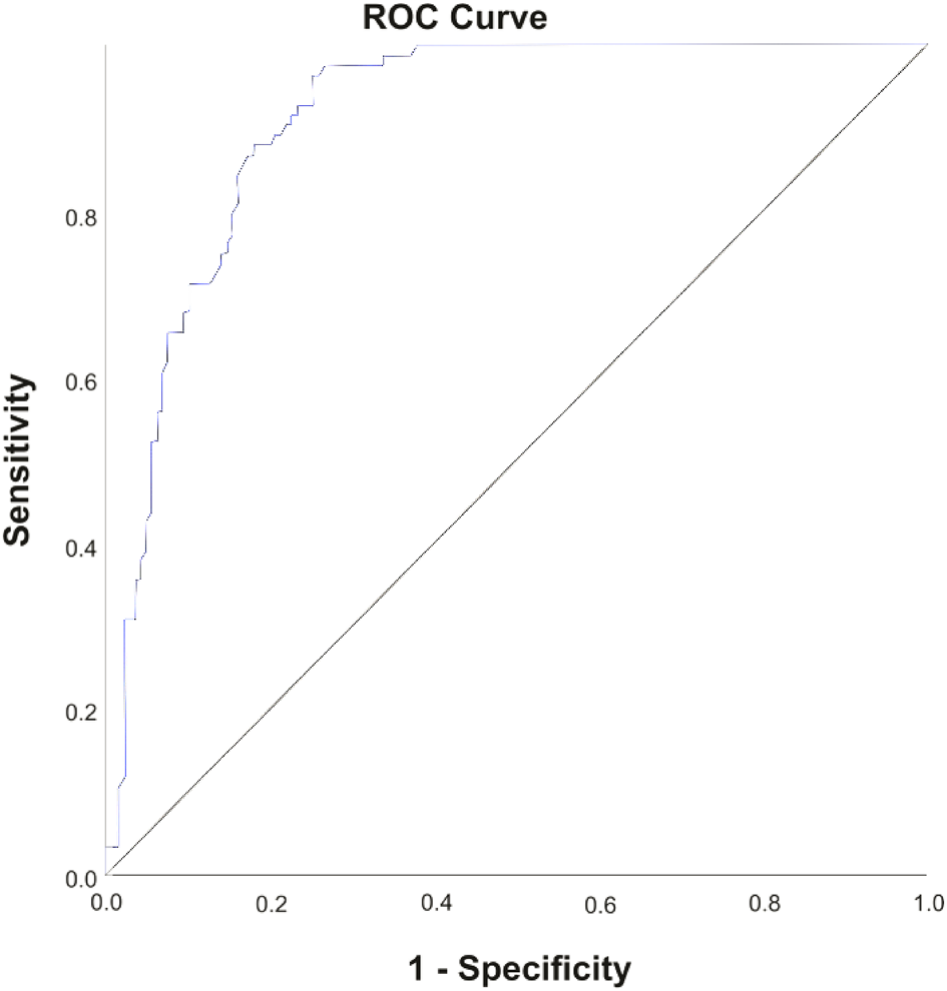
ROC analysis of cfDNA.

ROC analysis clearly illustrated that the curve for cfDNA was above the reference line thus further confirming the positive correlation of cfDNA with breast cancer (Fig. 2). Moreover, the analysis revealed that the concentration of cfDNA > 551.4 ng/µl (calculated by the Youden Index) could discriminate breast cancer patients from healthy controls, thus firmly declaring cfDNA a potent biomarker for breast cancer (Table 7).

**Table 7:** The area under the curve and optimum cut-off values for cfDNA in breast cancer patients.

AUC	Optimum cut-off Value	Sensitivity	Youden index	*P* value
0.915	551.4 ng/µl	0.964	0.714	< 0.001

*AUC* area under the curve.

## Discussion

Circulating cfDNA has been recognized for its clinical significance in various malignancies including breast cancer. In cancer patients, the production of larger cfDNA fragments due to processes such as necrosis results in elevated cfDNA levels compared to those in healthy individuals^30–33^. This study provides valuable insights into the potential of cfDNA as a diagnostic and prognostic biomarker in breast cancer patients. The researchers utilized Trucut biopsy and immunohistochemistry (IHC) markers to diagnose breast cancer cases, adding robustness to their findings. Through their investigation, significant associations were found between cfDNA levels and various clinicopathological characteristics and cancer biomarkers in patients with breast cancer. These outcomes conclude that cfDNA is a promising reliable indicator for diagnosing and assessing breast cancer prognosis.

One of the key findings was that the cfDNA levels were appreciably much more in breast cancer patients than in healthy controls. This observation aligns with previous research demonstrating elevated cfDNA levels in various cancer types, including breast cancer. For instance, Schwarzenbach et al. in 2011 reported that cfDNA levels were high in breast cancer patients' plasma compared to healthy controls and patients with benign breast diseases^34^. These consistent findings imply that cfDNA could serve as a noninvasive biomarker for early breast cancer detection and monitoring, offering a valuable tool for improving clinical management and patient outcomes.

Furthermore, the study revealed a significant association between cell-free DNA (cfDNA) levels and several cancer biomarkers, including estrogen receptor (ER), progesterone receptor (PR), human epidermal growth factor receptor 2 (HER2), triple-negative breast cancer (TNBC), and triple-positive breast cancer (TPBC). These findings are consistent with prior research that has demonstrated correlations between cfDNA levels and hormone receptor status in patients with breast cancer. For instance, a study conducted by Pushpanjali et al. in 2023 reported that elevated cfDNA levels are specifically associated with hormone receptor-positive breast cancer subtypes^35^. These results further support the notion that cfDNA analysis may offer valuable assistance in classifying and subtyping breast cancer, thereby aiding in more informed treatment decisions. By identifying the associations between cfDNA levels and various cancer biomarkers, this study contributes to using cfDNA as a helpful tool in breast cancer diagnosis and personalized treatment strategies.

Correlation analysis between cfDNA levels and clinical parameters yielded intriguing findings. Notably, hematological parameters such as hemoglobin (Hb), white blood cells (WBCs), and platelet count negatively correlated with cfDNA levels. In contrast, renal function test (RFT) parameters, specifically urea and creatinine levels, positively associated with cfDNA levels. These results suggest a potential association between cfDNA levels and kidney and liver functions. Although the precise mechanisms underlying these associations warrant further investigation, these findings emphasize the importance of considering the patient's overall health status when interpreting cfDNA results. Understanding the potential impact of kidney and liver function on cfDNA levels could have implications for using cfDNA as a diagnostic and prognostic biomarker in breast cancer patients, necessitating comprehensive clinical assessment to interpret cfDNA data accurately.

ROC analysis revealed that cfDNA has a high area under the curve (AUC) of 0.915, indicating its accuracy as a diagnostic and prognostic biomarker for breast cancer. With a sensitivity of 0.964, cfDNA demonstrated potential utility in effectively identifying patients with breast cancer. These findings align with a meta-analysis by Yu et al. (2019), which also reported the high sensitivity and specificity of cfDNA in diagnosing breast cancer. The determined optimal cut-off value of 551.4 ng/µl, using Youden's index provides a useful reference point for future studies and clinical applications of cfDNA in breast cancer detection and management^36^. Overall, cfDNA shows promise as a reliable and valuable tool for the diagnosis and prognosis of patients with breast cancer.

Despite its promising findings, this study has certain limitations that should be considered. First, the relatively small sample size may restrict the generalizability of the results to a broader population. Conducting larger cohort studies is essential to validate and strengthen the robustness of these findings. Second, this study did not explore the correlation between cfDNA levels and the stage and grade of breast cancer, which could offer valuable insights into disease severity and progression. Nonetheless, implementing cfDNA analysis in clinical practice can significantly improve early detection and enable personalized treatment strategies for patients with breast cancer, which could lead to better outcomes and management of the disease. Further research is warranted to fully understand the scope and benefits of cfDNA as a diagnostic and prognostic tool in breast cancer management.

## Conclusion

In conclusion, this study emphasizes the cfDNA as a promising biomarker for diagnosing breast cancer. The findings revealed elevated cfDNA levels in patients with breast cancer compared to healthy controls, indicating its relevance for early detection and disease monitoring. Furthermore, cfDNA correlates with various cancer biomarkers, aiding breast cancer classification. Despite these limitations, the high accuracy and sensitivity of cfDNA make it valuable for breast cancer diagnosis and personalized treatment strategies with the potential to enhance patient outcomes.

## Limitations

The key limitation of the current study was the limited sample size of 84 BC patients since we only included cases from a specific location. Applying limited techniques owing to cost restrictions was another limitation of this study.

### Supplementary Information

Supplementary Information.

## Data Availability

All of the data generated or collected during the whole research project has been provided in the manuscript.
