# Supplementary material for: Efficacy of cell-free DNA as a diagnostic biomarker in breast cancer patients
Source: Sci Rep. 2023 Sep 15;13:15347. doi: 10.1038/s41598-023-42726-6 (PMC10504267; doi:10.1038/s41598-023-42726-6)
Supplement: Supplementary file 1 — Supplementary Information. [file 41598_2023_42726_MOESM1_ESM.docx]

**Supplementary figure 1:** Pearson's correlation of clinical parameters with cfDNA in BC patients.
